# Proresolving mediator profiles in cerebrospinal fluid are linked with disease severity and outcome in adults with tuberculous meningitis

**DOI:** 10.1096/fj.201901590R

**Published:** 2019-10-05

**Authors:** Romain A. Colas, Le Thanh Hoang Nhat, Nguyen Thuy Thuong Thuong, Esteban A. Gómez, Lucy Ly, Hai Hoang Thanh, Nguyen Thi Hoang Mai, Nguyen Hoan Phu, Guy E. Thwaites, Jesmond Dalli

**Affiliations:** * William Harvey Research Institute, Barts and The London School of Medicine and Dentistry, Queen Mary University of London, London, United Kingdom;; † Oxford University Clinical Research Unit, Ho Chi Minh City, Vietnam;; ‡ Nuffield Department of Medicine, Centre for Tropical Medicine and Global Health, University of Oxford, Oxford, United Kingdom;; § Centre for Inflammation and Therapeutic Innovation, Queen Mary University of London, London, United Kingdom

**Keywords:** resolution, essential fatty acids, eicosanoids, aspirin, tuberculosis

## Abstract

Tuberculous meningitis (TBM) is the most lethal form of tuberculosis infection, characterized by a dysregulated immune response that frequently leads to neurologic injury and death despite the best available treatment. The mechanisms driving the inflammatory response in TBM are not well understood. To gain insights into these mechanisms, we used a lipid mediator–profiling approach to investigate the regulation of a novel group of host protective mediators, termed specialized proresolving mediators (SPMs), in the cerebrospinal fluid (CSF) of adults with TBM. Herein, using CSF from patients enrolled into a randomized placebo-controlled trial of adjunctive aspirin treatment, we found distinct lipid mediator profiles with increasing disease severity. These changes were linked with an up-regulation of inflammatory eicosanoids in patients with severe TBM and a decrease in the production of a number of SPMs. CSF proresolving mediator concentrations were also associated with 80-d survival. In survivors, we found a significant increase in proresolving mediator concentrations, including the lipoxygenase 5–derived 13-series resolvin (RvT)2, RvT4, and 15-epi-lipoxin B_4_, compared with those who died. Of note, treatment of patients with high-dose aspirin led to a decrease in the concentrations of the prothrombic mediator thromboxane A_2_, reduced brain infarcts, and decreased death in patients with TBM. Together, these findings identify a CSF SPM signature that is associated with disease severity and 80-d mortality in TBM.—Colas, R. A., Nhat, L. T. H., Thuong, N. T. T., Gómez, E. A., Ly, L., Thanh, H. H., Mai, N. T. H., Phu, N. H., Thwaites, G. E., Dalli, J. Proresolving mediator profiles in cerebrospinal fluid are linked with disease severity and outcome in adults with tuberculous meningitis.

*Mycobacterium tuberculosis* is responsible for more deaths globally than any other infectious disease. When it infects the brain and meninges to cause tuberculous meningitis (TBM), which represents 1–5% of all forms of tuberculosis, it either kills or severely disables around a half of all sufferers despite the best available treatment (1). The pathogenesis of TBM is not well understood, but poor outcomes have been linked to dysregulated intracerebral inflammation (2). Current therapeutic approaches are aimed at killing *M. tuberculosis* infecting the brain or controlling the inflammatory response (3, 4). To date, the latter has primarily involved adjunctive corticosteroid therapy and has been associated with increased survival, although how corticosteroids modulate intracerebral inflammation to influence outcomes remains uncertain (5).

It is now well established that under ideal conditions the body activates evolutionarily conserved programs to terminate inflammation and promote the repair and regeneration of damaged tissues (6). At the helm of these programs is a recently uncovered genus of lipid mediators produced *via* the stereoselective conversion of essential fatty acids and termed specialized proresolving mediators (SPMs). These mediators include the arachidonic acid (AA)-derived lipoxins (LXs), the eicosapentaenoic acid (EPA)-derived E-series resolvins (RvEs), the docosahexaenoic acid (DHA)-derived resolvins (RvDs), protectins (PDs), and maresins (MaRs), as well as the recently described n-3 docosapentaenoic acid (DPA)-derived resolvins (RvD_n-3 DPA_), protectins (PD_n-3 DPA_), and maresins (MaR_n-3 DPA_) (6). Recent studies demonstrate that SPMs regulate the phagocytosis and killing of bacteria during infections *via* the activation of cognate receptors. They counterregulate the production of proinflammatory mediators including cytokines and eicosanoids and control both leukocyte trafficking and phenotype (7–11). These autacoids also mediate the anti-inflammatory actions of several widely used therapeutics, including atorvastatin, pravastatin, and aspirin (7, 12, 13).

We recently tested the hypothesis that the addition of aspirin to standard TBM treatment (antituberculosis drugs and corticosteroids) may further improve outcomes by inhibiting thromboxane (Tx)A_2_, preventing brain infarcts (a common life-threatening complication of TBM) and enhancing the resolution of intracerebral inflammation through the increased expression of SPMs (14). The trial found that in patients with microbiologically confirmed TBM, aspirin was associated with reduced brain infarcts or death in the first 60 d of treatment.

Little is known about the regulation of lipid mediators, and in particular SPM, in the cerebrospinal fluid (CSF) during TBM. Thus, in the present studies, we investigated whether baseline CSF lipid mediator concentrations were altered with increasing disease severity. We found that concentrations of a number of SPM families were reduced with increasing disease severity. This was linked with an up-regulation of inflammation-initiating eicosanoids, including prostaglandins (PGs) and cysteinyl leukotrienes (LTs). Pretreatment CSF lipid mediator concentrations were also found to be predictive of outcome, with distinct lipid mediator profiles obtained in those patients that were alive at the end of the study *vs.* those that died during the study. Finally, we also found that in aspirin-treated patients there was a dose-dependent alteration of the CSF lipid mediator profiles.

## MATERIALS AND METHODS

### Ethics statement

The patients in the current study were enrolled in a clinical trial of adjunctive aspirin, the design and conduct of which have been previously described (14). Briefly, we conducted a parallel group, double-blind, randomized, placebo-controlled trial in HIV-uninfected adults with TBM to assess the safety and efficacy of either 81 or 1000 mg aspirin daily for the first 60 d of treatment with standard antituberculosis drugs and dexamethasone. The trial enrolled inpatients at the Hospital for Tropical Diseases, a 550-bed tertiary referral hospital in Ho Chi Minh City, Vietnam, and was approved by the Oxford Tropical Research Ethics Committee and the Institutional Review Board of the Hospital for Tropical Diseases and the Ethical Committee of the Ministry of Health (Vietnam). Adults (≥18 yr old) with suspected TBM (at least 5 d of meningitis symptoms, nuchal rigidity, and CSF abnormalities) and a negative HIV test were eligible to enter the trial. Written informed consent to participate in the study was obtained from all participants or from their relatives if the participant could not provide consent because of incapacity.

### Clinical study

Lumbar puncture was performed before the start of treatment and 30 d after treatment initiation, per normal clinical care with CSF archived at −80°C until later testing. Clinical progress and neurologic and drug-related adverse events were assessed daily until discharge from hospital and monthly thereafter until 8 mo, when a final clinical assessment was made.

### Targeted lipid mediator profiling

All samples were extracted using solid-phase extraction columns as previously described (15). Prior to sample extraction, deuterated internal standards, representing each region in the chromatographic analysis (500 pg each) were added to facilitate quantification in 4 vol of cold methanol. Samples were kept at −20°C for a minimum of 45 min to allow protein precipitation. Supernatants were subjected to solid-phase extraction and methyl formate and methanol fractions were collected, brought to dryness, and suspended in phase (methanol:water, 1:1, v/v) for injection on a Shimadzu LC-20AD HPLC and a Shimadzu SIL-20AC autoinjector (Kyoto, Japan), paired with a QTrap 5500 (Sciex, Framingham, MA, USA). For identification and quantitation of products eluted in the methyl formate, an Agilent Poroshell 120 EC-C18 Column (100 mm × 4.6 mm × 2.7 μm; Agilent Technologies, Santa Clara, CA, USA) was kept at 50°C, and mediators were eluted using a mobile phase consisting of methanol:water:acetic acid of 20:80:0.01 (v/v/v) that was ramped to 50:50:0.01 (v/v/v) over 0.5 min and then to 80:20:0.01 (v/v/v) from 2 to 11 min, maintained until 14.5 min, and then rapidly ramped to 98:2:0.01 (v/v/v) for the next 0.1 min. This was subsequently maintained at 98:2:0.01 (v/v/v) for 5.4 min, and the flow rate was maintained at 0.5 ml/min. QTrap 5500 was operated in negative ionization mode using a multiple reaction monitoring method as previously described (15).

In the analysis of peptide-conjugated lipid mediators eluting in methanol fraction, an Agilent Poroshell 120 EC-C18 column (100 mm × 4.6 mm × 2.7 μm) was kept at 50°C, and mediators were eluted using a mobile phase consisting of methanol:water:acetic acid at 55:45:0.1 (v/v/v) over 5 min that was ramped to 80:20:0.1 (v/v/v) for 2 min, maintained at 80:20:0.1 (v/v/v) for the next 3 min, and ramped to 98:2:0.1 (v/v/v) over 3 min. This was kept at 98:2:0.1 (v/v/v) for 3 min. A flow rate of 0.65 ml/min was used throughout the experiment. QTrap 5500 was operated in positive ionization mode using scheduled multiple reaction monitoring coupled with information-dependent acquisition and enhanced product ion scan (15).

Each lipid mediator was identified using established criteria, including matching retention time to synthetic and authentic materials and at least 6 diagnostic ions (15). Calibration curves were obtained for each using synthetic compound mixtures at 0.78, 1.56, 3.12, 6.25, 12.5, 25, 50, 100, and 200 pg, which gave linear calibration curves with *r*^2^ values of 0.98–0.99.

### Statistical analysis

We performed all statistical analyses and data derivation in R (*http://www.R-project.org/*) (16), Prism 8 (GraphPad, La Jolla, CA, USA), and Microsoft Excel (Microsoft, Redmond, WA, USA). Results are expressed as means and 95% confidence interval or interquartile range (IQR) as indicated in the figures and tables. Summary tables of the baseline characteristics of the study population with respect to disease severity, mortality outcome, and treatment allocation as median (IQR) for continuous data and *n* (%) for categorical data are provided in the supplemental document.

To assess the overall regulation of proinflammatory and proresolving mediator biosynthetic pathways in the CSF, we combined the concentrations of bioactive mediators from each of the lipid mediator families together with their biosynthetic pathway markers. For this purpose, we combined the concentrations of the RvDs (RvD1, RvD2, RvD3, RvD4, RvD5, RvD6, 17*R*-RvD1, and 17*R*-RvD3), PDs (PD1; 10*S*,17*S*-diHDHA; 17*R*-PD1; and 22-OH-PD1), protectin conjugate in tissue regeneration (PCTRs) (PCTR1, PCTR2, and PCTR3), and MaRs (MaR1; 7*S*,14*S*-diHDHA; MaR2; 4S,14*S*-diHDHA; and 22-OH-MaR1), maresins conjugated in tissue regeneration (MCTRs) (MCTR1, MCTR2, and MCTR3), the n-3 DPA-derived 13-series resolvins (RvTs) (RvT1, RvT2, RvT3, and RvT4), RvD_n-3 DPA_ (RvD1_n-3 DPA_, RvD2_n-3 DPA_, and RvD5_n-3 DPA_), PD_n-3 DPA_ (PD1_n-3 DPA_ and 10S,17*S*-diHDPA), and MaR_n-3 DPA_ (MaR1_n-3 DPA_ and 7*S*, 14*S*-diHDPA), the RvE (RvE1, RvE2, and RvE3), and the AA-derived LXs (LXA_4_; LXB_4_; 5S,15*S*-diHETE; 15-epi-LXA_4_; and 15-epi-LXB_4_). Separately, we combined the concentrations of proinflammatory eicosanoids: AA-derived LTs (LTB_4_; 5*S*,12*S*-diHETE; 12-epi-LTB_4_; 6-*trans*, 12-epi-LTB_4_; and 20-OH-LTB_4_), cysteinyl LTs (cysLTs) (LTC_4_, LTD_4_ and LTE_4_), PGs (PGD_2_, PGE_2_, and PGF_2α_), and Txs (TxB_2_).

Investigations into the flux down each of the mediator families was conducted by determining the fold change in the concentration of the different mediator families indicated previously, between the control groups [*i.e.*, modified British Medical Research Council criteria (MRC)1 or survivors] and test groups (*i.e.*, MRC2 + MRC3 or nonsurvivors, respectively).

Statistical differences between the concentrations (expressed as the log_2_ fold change) of the mediators in each analysis group was determined using Benjamini-Hochberg multiple testing correction to adjust for multiple testing, as implemented in R package “stats.” Lipid mediator networks were constructed using Cytoscape 3.7.1 (*https://cytoscape.org/download.html*), and the pathways that were statistically up- or down-regulated were denoted using red and blue lines, respectively.

Investigators were not blinded to group allocation or outcome assessment. The criterion for statistical significance was *P* ≤ 0.05. Partial least squares discriminant analysis (PLS-DA) and orthogonal PLS-DA (OPLS-DA) (17) were performed using Simca 14.1 software (Umetrics, Umea, Sweden) following mean centering and unit variance scaling of lipid mediator levels. PLS-DA is based on a linear multivariate model that identifies variables that contribute to class separation of observations (MRC scores, survivors/nonsurvivors; placebo/aspirin groups) on the basis of their variables (lipid mediator levels). During classification, observations were projected onto their respective class model. The score plot illustrates the systematic clusters among the observations (closer plots presenting higher similarity in the data matrix). Loading plot interpretation identified the variables with the best discriminatory power [variable importance in projection (VIP) >1] that were associated with the distinct intervals and contributed to the tight clusters comparisons of lipid mediators by disease severity (MRC grade) or mortality outcome. The Spearman test (18) was used to test for trend of ordinary groups for nonnormal continuous outcomes. The test was implemented in R package “compareGroups” (19). We adjusted for multiple testing for all the *P* values from all the comparisons based on Benjamini-Hochberg multiple testing correction. In addition, we also performed principal component analysis to assess whether the information in all lipid mediators can be simplified to a lower number of linear combinations, which helps to classify all the observations (MRC scores, survivors/nonsurvivors) on the basis of their variables (lipid mediator levels).

Furthermore, to identify the best subset of lipid mediators as predictors for disease severity and mortality outcomes in TBM, we performed a least absolute shrinkage and selection operator (LASSO) (20) regression model with 10-fold cross validation with 1000 iterations in which the disease severity or mortality was the outcome and all the lipid mediators were the covariates. This model was implemented in the R package “glmnet” (21).

Lipid mediator networks were constructed using Cytoscape 3.7.1, and the pathways that were statistically up- or down-regulated were denoted using red and blue lines, respectively.

The reduction of lipid mediators was computed as the difference between the 2 time points at baseline and at d 30. The reduction was compared between 2 pairs of treatment arms (81 mg aspirin *vs.* placebo and 1000 mg aspirin *vs.* placebo) based on a simple linear regression. In this model, the lipid mediator reduction is the outcome, and the treatment arm is the main covariate. To increase the power for the analysis, the model was also adjusted for the baseline lipid mediator. For indicated comparisons in this study, we performed multiplicity adjustments of *P* values for all comparisons based on Benjamin-Hochberg multiple testing correction.

## RESULTS

### Increased disease severity is associated with a reduction in baseline CSF SPM concentrations

**Figure 1:**
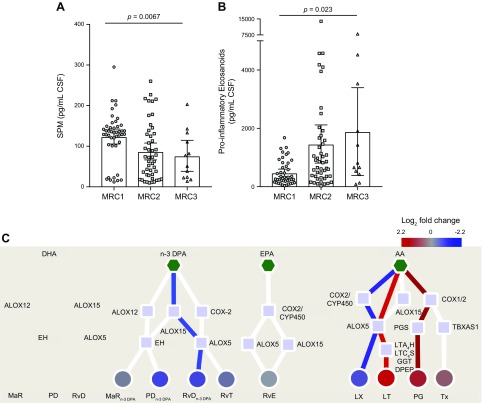
Reduced pretreatment CSF SPM concentrations prior to treatment initiation are associated with increased disease severity in TBM. CSF was collected from patients with TBM before the start of treatment, and lipid mediators were extracted, identified, and quantified using lipid mediator profiling. *A*) Sum of proresolving mediators (DHA-derived RvD, PD, PCTR, MaR, MCTR; n-3 DPA-derived RvT; RvD_n-3 DPA_, PD_n-3 DPA_, MaR_n-3 DPA_; EPA-derived RvE; AA-derived LX). *B*) Sum of proinflammatory eicosanoids (AA-derived LT, cysLT, PG, Tx). Results are means ± 95% confidence interval; *n* = 44 for MRC grade 1 (MRC1), *n* = 47 for MRC grade 2 (MRC2), and *n* = 12 for MRC grade 3 (MRC3). Statistical analysis was conducted using 1-way ANOVA followed by test for linear trend. *C*) Pathway interaction analysis down each of the lipid mediator families. Figure depicts the fold change, expressed as log_2_ fold change, in lipid mediator concentrations between patients with an MRC1 and those with MRC2 and MRC3. Scales represent fold increase or decrease for each mediator family. Mediator families colored in red or blue represent those families that were found to be significantly regulated. Statistical significance between mediator concentrations in patients with MRC1 and those with MRC2 and MRC3 grades was determined using unpaired Student’s *t* test and adjusted using Benjamini-Hochberg multiple testing correction. Red lines depict pathways that are up-regulated in patients with an MRC grade of 2 and 3 when compared with MRC1 patients. Blue lines depict pathways that are down-regulated in patients with an MRC2 + MRC3 when compared with MRC1 patients. Red circles depict lipid mediator families that are up-regulated in patients with an MRC2 + MRC3 when compared with MRC1 patients. Blue circles depict lipid mediator families that are down-regulated in patients with an MRC2 + MRC3 when compared with MRC1 patients. Pentagons depict the distinct essential fatty acids, squares the lipid mediator biosynthetic enzymes, and circles the distinct lipid mediator families. CYP450, cytochrome P450; DPEP, dipeptidase; EH, epoxide hydrolase; GGT, γ-glutamylt ransferase; GSTM, glutathione *S*-transferase Mu; LTA_4_H, LTA_4_ hydrolase; LTC_4_S, LTC_4_ synthase; PGS, PG synthase; TBXAS1, TxA synthase.

In order to determine whether there was a relationship between CSF lipid mediator concentrations and disease severity in TBM, we investigated baseline lipid mediator profiles of patients recruited to the aspirin TBM study (NCT02237365) (14). Here, CSF was collected from enrolled patients with known or suspected TBM just prior to the start of treatment in a randomized comparison of aspirin *vs.* placebo as an adjunct to dexamethasone administration for the first 60 d of TBM treatment (14). Disease severity was assessed using the MRC grade (22), which uses Glasgow coma score and focal neurologic deficits to categorize disease severity as mild (grade 1), moderate (grade 2), or severe (grade 3). Out of the total 120 patients, we only had sufficient plasma from 105 patients, and a further 2 patients were excluded from the analysis because these were found to be negative for TBM, leaving a total of 103 patients (See Supplemental Table S1 for patient information). Using targeted liquid chromatography-tandem mass spectrometry–based profiling of CSF, we identified mediators from all 4 major bioactive metabolomes, including the AA-derived (LXs, LTs, and PGs) and the n-3 DPA-, EPA-, and DHA-derived resolvins (Supplemental Table S2). In order to gain insights into the relationship between baseline CSF lipid mediator concentrations and disease severity, we first grouped the mediators by biologic function, assessing the differences between proresolving mediators (LXs, resolvins, protectins, and maresins) and proinflammatory mediators (LTs, PGs, and Txs) in patients with moderate or severe disease (MRC grades 2 and 3) when compared with those with mild disease (MRC grade 1) (23) (**Fig. 1*A*** and Supplemental Table S2). Results of this analysis demonstrated that with increasing disease severity there was a decrease in overall proresolving lipid mediator concentrations in the CSF of patients with TBM (Fig. 1*A*). This relationship was coupled with an increase in the concentrations of proinflammatory mediators in those with more severe disease (Fig. 1*B*).

In order to gain further insights into the mediator pathways that were differentially regulated between these patient groups, we next interrogated the biosynthetic pathways for each of the essential fatty acid metabolomes. This analysis demonstrated that in patients with an MRC score of 2 and 3, when compared with patients with an MRC score of 1, there was a significant reduction in 2 proresolving mediator families, the RvD_n-3 DPA_ and the AA-derived LXs. This was coupled with a significant increase in the proinflammatory AA-derived PGs and LTs (Fig. 1*C*).

**Figure 2:**
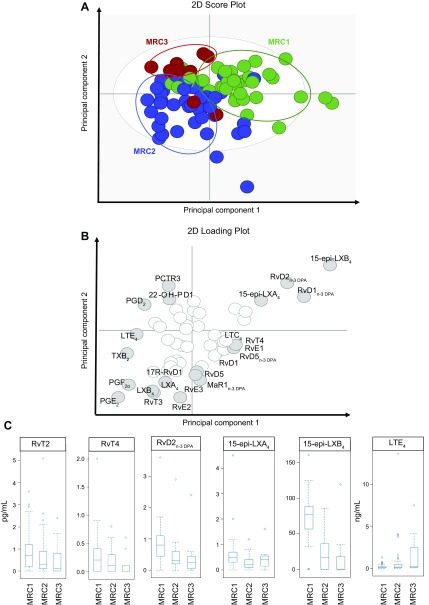
TBM disease severity is linked with a switch in the ALOX product profile. CSF was collected from patients with TBM before the start of treatment, and lipid mediators were extracted, identified, and quantified using lipid mediator profiling. Differences in lipid mediator profiles between the 3 groups were evaluated using PLS-DA. *A*) Score plot depicting the distribution of individual patients from each of the 3 MRC groups. Green circles depict mediator profiles from MRC1 patients, blue circles depict mediator profiles from MRC2 patients, and red circles depict lipid mediator profiles from MRC3 patients. *B*) Corresponding loading plot. Gray circles depict mediators with VIP scores >1. *C*) Box-plot of lipid mediators found to be significantly associated with disease severity; *n* = 44 for MRC1, *n* = 47 for MRC2, and *n* = 12 for MRC3. 2D, 2-dimensional.

We next employed PSL-DA, a regression model that identifies variables that contribute to the separation of experimental groups, to investigate the relationship between lipid mediator profiles (*i.e.*, the concentrations of individual mediators identified in the CSF) and disease severity. This analysis demonstrated that the individual lipid mediator concentrations were markedly different between patients with MRC grades 1, 2, and 3, as demonstrated by the distinct clustering of patients from the different disease severity groups (**Fig. 2*A***). Assessment of the VIP scores, which identify the contribution of each mediator in the observed separation between each of the groups, identified 24 mediators that had a VIP score >1, showing that they were distinctly regulated depending on disease severity (Fig. 2*B*). Among the mediators found to be differentially regulated between these 3 disease groups were several lipoxygenase (ALOX)5-derived mediators that are involved in coordinating the host response to clear bacterial infections, including RvT4 and RvE1 (Fig. 2*B*) (7, 24, 25).

Having identified that there was a differential regulation of lipid mediator profiles with increasing disease severity, we next assessed the association between distinct lipid mediators and disease severity. There was a significant negative correlation between a select group of proresolving mediators, 15-epi-LXB_4_, RvD2_n-3 DPA_, 22-OH-PD1, MaR1, and 15-epi-LXA_4_, and increasing disease severity (Fig. 2*C* and Supplemental Table S2). In addition, we also observed increased concentrations of LTE_4_, the terminal product in the cysLT biosynthetic metabolome that was recently found to be also bioactive (26), with increasing disease severity, although the correlation was not statistically significant after correction for multiple testing (Fig. 2*C* and Supplemental Table S2).

In order to designate the best subset of lipid mediators as predictors of the disease severity, we conducted multiple regression analysis on mediators that were found to be differentially expressed between the patient groups. For this purpose, we used LASSO regression analysis. We identify a subset of 20 lipid mediators that are predictors of disease severity (Supplemental Table S3). Among them, 4 lipid mediators (15-epi-LXB_4_, LXB_4_, PGE_2_, 22-OH-PD1) gave the strongest predictive value, whereby decreased concentrations of these proresolving mediators were associated with increased disease severity. Furthermore, 2 of the lipid mediators, RvD2_n-3 DPA_ and 15-epi-LXB_4_, identified by univariate analysis, were validated by LASSO regression with high frequencies in disease severe prediction (Supplemental Table S3).

Having established that SPM concentrations are differentially regulated, we next sought to assess whether the down-regulation observed in SPM production was due to a reduction in the concentrations of free essential fatty acid substrates involved in the biosynthesis of SPMs. Concentrations of DHA, n-3 DPA, EPA, and AA were all increased with increasing disease severity (Supplemental Fig. S1*A*). We next assessed whether SPM biosynthetic enzyme activity was altered in CSF from these patients. For this purpose, we measured the concentrations of monohydroxylated fatty acids that are produced by each of the enzymes. Assessment ALOX15 products 17-docosahexaenoic acid (HDHA), 17-hydroxydocosapentaenoic acid (HDPA), 15-hydroxyicosapentaenoic acid (HEPE), and 15-hydroxyeicosatetraenoic acid (HETE) indicated a significant up-regulation of ALOX15 activity with increasing disease severity because the concentrations of these products were significantly increased with increasing disease severity (Supplemental Fig. S1*B*). Concentrations of ALOX12, ALOX5, and cyclooxygenase (COX)-derived monohydroxylated products did not significantly change, suggesting that the activity of these enzymes was not significantly altered between the different patient groups (Supplemental Fig. S1*B*).

Given the role that immune cells play in the biosynthesis of lipid mediators (7, 9), we next investigated whether disease severity influenced CSF leukocyte numbers. Here, we found that CSF leukocyte numbers were inversely correlated with increasing disease severity, primarily linked with a reduction in neutrophil counts (Supplemental Fig. S2). We next assessed whether there was an association between CSF leukocyte counts and the concentrations for each of the identified lipid mediator families. With adjustment for multiple testing, no significant correlations were found between these parameters (unpublished results), suggesting that the observed difference may be due to a differential leukocyte activation.

### Pretreatment SPM and eicosanoid concentrations in CSF correlate with mortality

**Figure 3:**
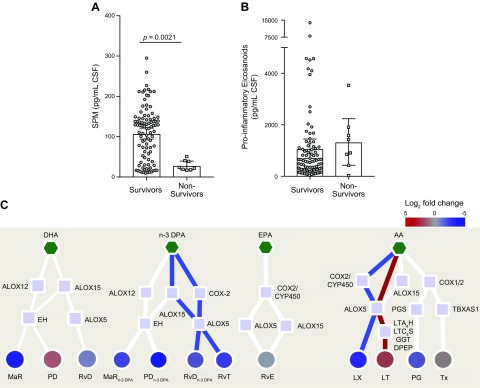
Pretreatment CSF resolution status is associated with poor outcome from TBM. CSF was collected before the start of treatment, and lipid mediators were identified and quantified using lipid mediator profiling. *A*) Sum of proresolving mediators. *B*) Sum of proinflammatory eicosanoids. Statistical analysis was conducted using 1-way ANOVA with Dunnet *post hoc* test. Results are means, with error bars depicting minimum to maximum values; *n* = 95 survivors and 8 nonsurvivors. *C*) Interaction networks were constructed to compare the pretreatment CSF lipid mediator profiles from patients that died during the 80-d trial period to those that survived. Scales represent fold increase or decrease for each mediator family. Mediator families colored in red or blue represent those families that were found to be significantly regulated. Statistical significance was determined using unpaired *t* test and adjusted using Benjamini-Hochberg multiple testing correction. Results are representative of *n* = 8 patients. Red lines depict pathways that are up-regulated in nonsurvivors. Blue lines depict pathways that are down-regulated in nonsurvivors. Red circles depict lipid mediator families that are up-regulated in nonsurvivors. Blue circles depict lipid mediator families that are down-regulated in nonsurvivors. Pentagons depict the distinct essential fatty acids, squares the lipid mediator biosynthetic enzymes, and circles the distinct lipid mediator families. DPEP, dipeptidase; EH, epoxide hydrolase; GGT, γ-glutamyl transferase.

Having found that SPM concentrations were associated with TBM disease severity, we investigated whether CSF lipid mediator concentrations were also linked with mortality. The demographics and baseline characteristics of study population by disease outcome at baseline (MRC grade) are summarized in Supplemental Table S4. We first assessed the CSF concentrations of SPM and proinflammatory eicosanoids, finding that SPM concentrations were significantly reduced in patients that died during the study, whereas proinflammatory mediator concentrations tended to be higher in those who survived, although this did not reach statistical significance (**Fig. 3*A, B*** and Supplemental Table S5).

We next conducted lipid mediator biosynthetic pathway analysis to evaluate which pathways are contributing to the observed differences in lipid mediator concentrations. This demonstrated that there was a down-regulation in the expression of RvT, RvD_n-3 DPA_, and LX, with an up-regulation in the proinflammatory LT in nonsurvivors when compared with survivors (Fig. 3*C*).

**Figure 4:**
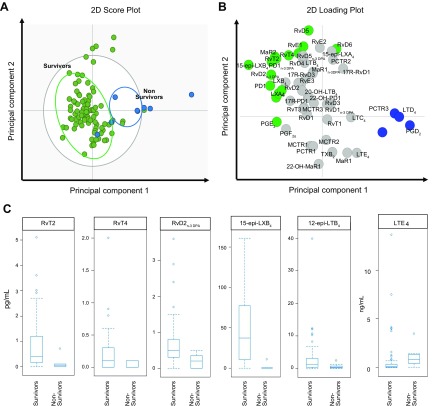
Death from TBM is associated with a down-regulation of CSF ALOX5-derived SPM and an up-regulation of ALOX5-derived LTE_4_. CSF lipid mediator profiles obtained from patients that died during the 80-d duration of the study (nonsurvivors) were compared with those that were alive at the end of the study (survivors) using OPLS-DA. *A*) Score plot. Green circles represent lipid mediator profiles from survivors, and blue circles represent lipid mediator profiles from nonsurvivors. *B*) Loading plot. Green circles and blue circles represent lipid mediators with VIP scores >1 and their link with either survivors or nonsurvivors based on their distribution in their loading plot. *C*) Box-plot of lipid measurement *vs.* 80-d mortality outcome of 6 lipid mediators, which are significantly associated with mortality; *n* = 95 survivors and 8 nonsurvivors. 2D, 2-dimensional.

OPLS-DA analysis of CSF lipid mediator profiles provided further support for the differences in lipid mediator concentrations between those who died and those who survived, with CSF lipid mediator concentrations from each of these patient groups giving 2 distinct clusters (**Fig. 4*A***). This separation between the 2 groups was linked with a differential regulation of 18 lipid mediators, which gave a VIP score >1 and included MaR2, RvT2, and 15-epi-LXB_4_. Of note, RvT2 and 15-epi-LXB_4_ activate the innate immune response to clear bacterial infections and counterregulate the production of proinflammatory mediators (6, 7). Statistical assessment of mediators found to be differentially regulated demonstrated that the concentrations of RvT2 and 15-epi-LXB_4_ were significantly lower in nonsurvivors when compared with survivors (Fig. 4*B* and Supplemental Table S5). Out of these mediators, 15-epi-LXB_4_ with median and IQR in 2 groups [survivor: 37.5 (10.2; 77.3); death 0.00 (0.00; 0.20), adjusted *P* = 0.02)] was found to be significantly associated with the mortality after adjustment for multiple testing (Fig. 4*C* and Supplemental Table S5). We next tested whether lipid mediators found to be differentially regulated between the 2 groups were correlated with outcome. Using LASSO regression analysis, we found that CSF concentrations of 15-epi-LXB_4_ together with those of the immunosuppressive mediator PGD_2_ were the stronger predictors of mortality (Supplemental Table S6).

In order to gain insights into the mechanisms contributing to the differences in CSF lipid mediator concentrations in TBM survivors and nonsurvivors, we next investigated whether the concentrations of essential fatty acids that are substrates in the biosynthesis of these molecules were different between these 2 patient groups. Of note, CSF concentrations of all fatty acids were significantly higher in nonsurvivors when compared with survivors (Supplemental Fig. S3). We next queried whether activity of the SPM biosynthetic enzymes was reduced in nonsurvivors, thus potentially accounting for the reduced SPM levels in the CSF of these patients. Assessment of monohydroxylated fatty acid products of these enzymes indicated that the activity of all 4 enzymes was significantly up-regulated in nonsurvivors when compared with survivors (Supplemental Fig. S3). Thus, these findings demonstrate that the reduction in SPM concentrations was not due to either limited substrate availability of reduced activity of the biosynthetic enzymes.

### Aspirin administration up-regulates CSF concentrations of select proresolving mediators during TBM

**Figure 5:**
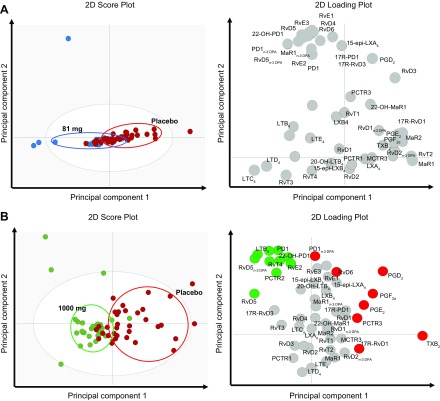
Administration of 1000 mg/d of aspirin markedly alters CSF lipid mediator profiles after 30 d of treatment. CSF fluids were collected 30 d after administration of 81 or 1000 mg aspirin per day or placebo. Lipid mediators were extracted, identified, and quantified using lipid mediator profiling. Differences between CSF lipid mediator profiles of patients administered 81 (*A*) or 1000 (*B*) mg aspirin per day in comparison with those given placebo were evaluated using OPLS-DA. (Left panel) score plot; (right panel) loading plot. Mediators with a VIP score >1 are identified in red (placebo), green (1000 mg aspirin), or blue (81 mg aspirin) circles that denote the association with the placebo and aspirin group. Results are representative of *n* = 28 for patients in the 81-mg aspirin group, *n* = 27 patients in the 1000-mg aspirin group, and *n* = 34 patients in the placebo group. 2D, 2-dimensional.

We recently reported that treatment with aspirin, dexamethasone, and antituberculosis drugs was associated with reduction in new brain infarcts and deaths within 60 d in patients with microbiologically confirmed TBM (14). Therefore, we investigated the impact of aspirin cotreatment with dexamethasone and antituberculosis drugs on CSF lipid mediator pathways in this patient subpopulation (see Supplemental Table S7 for demographics and baseline characteristics). We compared lipid mediator profiles obtained after 30 d of aspirin (1000 or 81 mg/d) or placebo (Supplemental Table S7) cotreatment. OPLS-DA analysis demonstrated that, whereas d-30 CSF lipid mediator profiles between the 81-mg and placebo groups were not markedly different, lipid mediator profiles from patients given 1000 mg aspirin gave distinct clusters compared with patients given placebo (**Fig. 5*A, B***). This separation between the 2 patient groups was linked with a differential regulation of 18 mediators from all the 4 bioactive metabolomes, including RvT4 and TxB_2_, the inactive breakdown product of the prothrombotic TxA_2_ (27) (Fig. 5*B*).

We next conducted statistical analysis to assess whether cotreatment with aspirin changes lipid mediator profiles between baseline and d 30 when compared with placebo. For this analysis, we started with 93 patients for whom we had matched samples for lipid mediator profiles at d 0 and 30. We excluded 13 patients who received aspirin for <30 d and 1 patient who was found to be a statistical outlier for lipid mediator concentrations. This left a total of 79 patients that were included in the analysis (placebo *n* = 29 *vs.* 2 doses: 1000 mg aspirin *n* = 23, 81 mg aspirin *n* = 27). Assessment of CSF lipid mediator concentrations demonstrated a reduction in TxB_2_ concentrations [mean and 95% confidence interval per treatment group; placebo 16.2 (6.97; 30.6) and 1.10 (0.50; 2.00)], the inactive further metabolite of the immunosuppressive, and prothrombotic mediator TxA_2_. (Supplemental Tables S8 and S9). The reduction in TxB_2_ concentrations was found to reach statistical significance at d 30 posttreatment initiation in patients given 1000 mg aspirin after adjustment for multiple testing (adjusted *P* < 0.001; Supplemental Table S9).

## DISCUSSION

In the present study, we investigated the regulation of CSF lipid mediator profiles before and during the treatment of adults with TBM. We found that pretreatment disease severity was associated with the concentrations of both inflammatory and proresolving mediators, with more severe disease linked to lower SPM concentrations and increased concentrations of immunosuppressive, vasoconstrictive, and nociceptive eicosanoids. Pretreatment SPM concentrations were also associated with 80-d mortality, with survivors having higher concentrations of several SPM families, including the ALOX-derived Rvs and LXs, compared with those who died. Aspirin coadministration with dexamethasone was observed to increase the CSF resolution index after 30 d of treatment and decrease TxB_2_ concentrations.

Recent decades have seen significant advances in our ability to manage patients with TBM; however, morbidity and mortality remain high (1). This is at least partly due to our limited understanding of the underlying mechanisms that perpetuate inflammation within the CNS leading to disability and ultimately death despite the best available treatment. In the present study, we investigated the relationship between disease severity and local mediator concentrations. Results from these analyses demonstrate that even prior to the initiation of treatment, patients that died during the course of the study presented with a profound dysregulation in both protective and inflammatory mediator pathways.

Lipid mediator biosynthesis is a tightly coordinated process in which essential fatty acids are sequentially oxygenated, primarily by ALOX and COX enzymes, to produce stereochemically defined and structurally unique products (6, 28). Regulation of these biosynthetic enzymes occurs at both a transcriptional/translational level as well as *via* post-translational modifications. Recent studies demonstrate that NOS promotes the *S*-nitrosylation of COX-2, increasing its catalytic activity and up-regulating the production of protective mediators including PGI_2_ (29) and RvT (7). On the other hand, the calcium-calmodulin-dependent protein kinase II-p38-mediated phosphorylation of ALOX5 leads to the translocation of the enzyme from the cytosol, where it is coupled with ALOX15 to produce SPM, to the nuclear membrane. At the nuclear membrane, this enzyme couples with phospholipase A_2_ and LTA_4_ hydrolase to produce LTB_4_ (10, 30) or LTC_4_ synthase to produce cysLTs. These mediators play complementary roles in amplifying the immune response in which LTC_4_ promotes vascular leakage and edema formation, whereas LTB_4_ is a potent chemoattractant to recruit new cells from the circulation (28). In the present study, we found that with increasing disease severity, as well as in patients that did not survive, there was a decrease in the concentrations of ALOX15/ALOX5-derived SPMs and a concomitant up-regulation in LT production. Of note, this reduction in SPMs was not linked with either a reduction in substrate availability or biosynthetic enzyme activity given that both of these parameters were found to be either identical or up-regulated in nonsurvivors. Thus, these findings suggest that disease severity may be linked with a decoupling of SPM biosynthetic enzymes and a concomitant up-regulation of proinflammatory pathways.

Leukocytes play an important role in the biosynthesis of lipid mediators, with their product profile reflecting their activation status (31–33). Different macrophage subsets, for example, display distinct lipid mediator profiles, with monocyte-derived macrophages skewed toward a classic phenotype expressing higher amounts of proinflammatory eicosanoids, whereas cells with an alternatively activated phenotype display higher concentrations of proresolving mediators (31–33). This shift is also linked with a differential expression of both lipid mediator biosynthetic enzymes as well as the phosphorylation status of ALOX5 (32–34). In the present study we found a decrease in the number of leukocytes in the CSF with increasing disease severity. However, there was no correlation between leukocyte numbers and lipid mediator concentrations, suggesting that regulation of enzyme activity, possibly reflecting a shift in leukocyte phenotype or population, is responsible for the altered lipid mediator concentrations. Future studies will need to further investigate the mechanisms leading to the observed changes in LM profiles in these patients, assessing aspects such as subcellular localization of the SPM biosynthetic enzymes, as well as the expression of these proteins in cells of the CNS. Another aspect that would need to be taken into consideration is the activation status of cells, in particular leukocytes recruited into the CSF, given that a better understanding of these cellular and molecular mechanisms may provide novel therapeutic opportunities in TBM.

Studies conducted by Vane *et al*. (35) demonstrate that aspirin inhibits the production of PGs and Tx, a mechanism that is dependent on the acetylation of COX-1 and COX-2. Later investigations by Serhan *et al*. (36, 37) found that acetylation of COX-2 also led to a switch in the catalytic activity of the enzyme, from the production of PGG_2_ to the formation of the precursor in the biosynthesis of epimeric forms of resolvins, LXs, and protectins. These precursors are 15R-hydropreroxy-eicosatetraneoic acid precursor in the biosynthesis of aspirin-triggered LXs and 17R-hydroperoxy-docosanexaenoic precursor in the biosynthesis of aspirin-triggered resolvins and protectins. In the present study, we found that high-dose aspirin administration reduced TxB_2_ concentrations and was linked with improved outcomes, whereas concentrations of aspirin-triggered–SPM were comparable between the 3 groups. The observation that aspirin-triggered–SPM concentrations were not changed by aspirin treatment may be because the present study was not adequately powered for this analysis or because, at the interval tested (*i.e.*, 30 d postinitiation of treatment), the biosynthetic pathways leading to the formation of these molecules were down-regulated. Future studies powered to interrogate this question will need to establish whether daily administration of high-dose aspirin down-regulates these pathways in the CSF.

In summary, the present findings uncover novel mechanisms in the pathophysiology of TBM that may have relevance to all forms of tuberculosis. Disease severity is associated with a differential expression of lipid mediators that includes a reduction in the concentrations of several SPM, an observation that is also linked with a poor prognosis.

## Supplementary Material

This article includes supplemental data. Please visit *http://www.fasebj.org* to obtain this information.

## Abbreviations

AA: arachidonic acid
ALOX: lipoxygenase
COX: cyclooxygenase
CSF: cerebrospinal fluid
cysLT: cysteinyl LT
DHA: docosahexaenoic acid
DPA: docosapentaenoic acid
EPA: eicosapentaenoic acid
IQR: interquartile range
LASSO: least absolute shrinkage and selection operator
LT: leukotriene
LX: lipoxin
MaR: DHA-derived maresin
MaR_n-3 DPA_: n-3 DPA-derived maresin
MCTR: maresin conjugated in tissue regeneration
MRC: modified British Medical Research Council criteria
OPLS-DA: orthogonal PLS-DA
PCTR: protectin conjugate in tissue regeneration
PD: DHA-derived protectin
PD_n-3 DPA_: n-3 DPA-derived protectin
PG: prostaglandin
PLS-DA: partial least squares discriminant analysis
RvD: DHA-derived resolvin
RvD_n-3 DPA_: n-3 DPA-derived resolvins
RvE: EPA-derived E-series resolvin
RvT: 13-series resolvin
SPM: specialized proresolving mediator
TBM: tuberculous meningitis
Tx: thromboxane
VIP: variable importance in projection
