# Supplementary material for: Proresolving mediator profiles in cerebrospinal fluid are linked with disease severity and outcome in adults with tuberculous meningitis
Source: FASEB J. 2019 Oct 5;33(11):13028–39. doi: 10.1096/fj.201901590R (PMC6902685; doi:10.1096/fj.201901590R)
Supplement: Supplementary file 1 [file fj.201901590R.sd1.pdf]

Pro-Resolving Mediator Profiles In Cerebrospinal Fluid are Linked with Disease Severity and Outcome in Adults with Tuberculous Meningitis

Romain A. Colas[1*], Le Thanh Hoang Nhat[2*], Nguyen Thuy Thuong Thuong[2], Esteban A. Gómez[1], Lucy Ly[1], Hai Hoang Thanh[2], Nguyen Hoan Phu[2], Nguyen Thi Hoang Mai[2], Guy E. Thwaites[2,3,$] and Jesmond Dalli[1,4,$,+].

[1]William Harvey Research Institute, Barts and The London School of Medicine and Dentistry, Queen Mary University of London, Charterhouse Square, London, EC1M 6BQ UK. [2]Oxford University Clinical Research Unit, Ho Chi Minh City, Vietnam [3]Centre for Tropical Medicine and Global Health, Nuffield Department of Medicine, University of Oxford, Oxford, UK[4] Centre for Inflammation and Therapeutic Innovation, Queen Mary University of London, London, UK.

Supplemental Tables

Supplemental Table 1: Summary data set MRC1, MRC2, MRC3

Characteristic	n	Summary statistic #MRC1 (N=44)	n	Summary statistic MRC2 (N=47)	n	Summary statistic MRC3 (N=12)
Gender – no. (%)	44		46		12	
- Male		22/44 (50%)		34/46 (74%)		8/12 (67%)
- Female		22/44 (50%)		12/46 (26%)		4/12 (33%)
Age (years) – median (IQR)	44	38.00(27.75,50.25)	46	42.50(32.25,49.00)	12	40.00(37.25,45.50)
Weight (kg) – median (IQR)	44	52.00(45.00,61.25)	46	50.00(46.00,58.00)	12	55.75(45.38,60.50)
Glasgow coma score – median (IQR)	44	15.00(15.00,15.00)	47	13.00(12.00,14.00)	12	8.50(8.00,10.00)
*Diagnostic category – no. (%)	44		47		12	
- definite TBM		33/44 (75%)		40/47 (85%)		8/12 (67%)
- possible TBM		9/44 (20%)		5/47 (11%)		1/12 (8%)
- probable TBM		2/44 (5%)		2/47 (4%)		3/12 (25%)
Treatment arm – no. (%)	44		47		12	
- Aspirin_1000mg		15/44 (34%)		13/47 (28%)		4/12 (33%)
- Aspirin_81mg		14/44 (32%)		16/47 (34%)		5/12 (42%)
- Placebo		15/44 (34%)		18/47 (38%)		3/12 (25%)

All summary statistics are absolute counts (%) for categorical variables and median (inter-quartile range = IQR) for continuous data. n refers to the number of patients with non-missing data for the corresponding variable.
#MRC denotes modified British Medical Research Council criteria. MRC1 indicates a GCS of 15 with no neurologic signs (baseline), MRC2 a score of 11 to 14 (or 15 with focal neurologic signs), and MRC3 a score of 10 or less.
*Diagnostic categories were assigned according to the consensus case definition (Marais et al.; The Lancet Infectious diseases. 2010; **10**(11): 803-12).

Supplemental Table 2: Comparison of lipid mediators by disease severity (MRC grade)

Lipid Mediators		1 (N=44)	2 (N=47)	3 (N=12)	P-value	Adjusted p-value
**DHA metabolome**						
RvD1	Negative*	27 (61.4%)	30 (63.8%)	7 (58.3%)	0.972	0.993
	Positive	17 (38.6%)	17 (36.2%)	5 (41.7%)		
RvD2	Negative	27 (61.4%)	33 (70.2%)	7 (58.3%)	0.802	0.925
	Positive	17 (38.6%)	14 (29.8%)	5 (41.7%)		
RvD3	Negative	33 (75.0%)	31 (66.0%)	10 (83.3%)	0.997	0.998
	Positive	11 (25.0%)	16 (34.0%)	2 (16.7%)		
RvD4		0.30 [0.10;0.43]	0.20 [0.10;0.55]	0.20 [0.00;0.40]	0.252	0.492
RvD5		0.70 [0.20;1.07]	0.40 [0.15;1.00]	0.25 [0.00;0.80]	0.121	0.356
RvD6	Negative	31 (70.5%)	30 (63.8%)	7 (58.3%)	0.374	0.577
	Positive	13 (29.5%)	17 (36.2%)	5 (41.7%)		
17R-RvD1	Negative	26 (59.1%)	23 (48.9%)	3 (25.0%)	0.045**	0.22
	Positive	18 (40.9%)	24 (51.1%)	9 (75.0%)		
17R-RvD3	Negative	26 (59.1%)	33 (70.2%)	11 (91.7%)	0.034**	0.204
	Positive	18 (40.9%)	14 (29.8%)	1 (8.33%)		
PD1		0.30 [0.00;0.40]	0.30 [0.00;0.50]	0.10 [0.00;0.23]	0.959	0.993
10S,17S-diHDHA		0.10 [0.00;0.52]	0.40 [0.10;0.70]	0.10 [0.00;0.52]	0.197	0.425

17R-PD1	Negative	41 (93.2%)	42 (89.4%)	10 (83.3%)	0.297	0.534
	Positive	3 (6.82%)	5 (10.6%)	2 (16.7%)		
22-OH-PD1	Negative	42 (95.5%)	42 (89.4%)	5 (41.7%)	<0.001**	0.001
	Positive	2 (4.55%)	5 (10.6%)	7 (58.3%)		
PCTR1	Negative	39 (88.6%)	41 (87.2%)	11 (91.7%)	0.901	0.993
	Positive	5 (11.4%)	6 (12.8%)	1 (8.33%)		
PCTR2	Negative	40 (90.9%)	45 (95.7%)	12 (100%)	0.181	0.408
	Positive	4 (9.09%)	2 (4.26%)	0 (0.00%)		
PCTR3	Negative	34 (77.3%)	38 (80.9%)	7 (58.3%)	0.394	0.59
	Positive	10 (22.7%)	9 (19.1%)	5 (41.7%)		
MaR1	Negative	34 (77.3%)	24 (51.1%)	5 (41.7%)	0.005**	0.05
	Positive	10 (22.7%)	23 (48.9%)	7 (58.3%)		
7S-14S-diHDHA	Negative	27 (61.4%)	34 (72.3%)	7 (58.3%)	0.727	0.921
	Positive	17 (38.6%)	13 (27.7%)	5 (41.7%)		
MaR2		2.70 [0.10;12.8]	1.30 [0.15;15.0]	0.45 [0.18;0.88]	0.255	0.492
4S,14S-diHDHA	Negative	22 (50.0%)	32 (68.1%)	8 (66.7%)	0.115	0.356
	Positive	22 (50.0%)	15 (31.9%)	4 (33.3%)		
22-OH-MaR1	Negative	30 (68.2%)	31 (66.0%)	5 (41.7%)	0.169	0.398
	Positive	14 (31.8%)	16 (34.0%)	7 (58.3%)		
MCTR1	Negative	42 (95.5%)	43 (91.5%)	11 (91.7%)	0.494	0.667
	Positive	2 (4.55%)	4 (8.51%)	1 (8.33%)		
MCTR2	Negative	44 (100%)	45 (95.7%)	11 (91.7%)	0.092	0.311
	Positive	0 (0.00%)	2 (4.26%)	1 (8.33%)		
MCTR3	Negative	38 (86.4%)	40 (85.1%)	11 (91.7%)	0.781	0.925
	Positive	6 (13.6%)	7 (14.9%)	1 (8.33%)		
**n-3 DPA metabolome**						
RvT1		0.15 [0.10;0.23]	0.10 [0.00;0.25]	0.10 [0.10;0.20]	0.329	0.568
RvT2		0.70 [0.20;1.20]	0.30 [0.10;0.90]	0.10 [0.00;0.50]	0.015**	0.136
RvT3	Negative	26 (59.1%)	23 (48.9%)	9 (75.0%)	0.763	0.925
	Positive	18 (40.9%)	24 (51.1%)	3 (25.0%)		
RvT4		0.20 [0.00;0.40]	0.10 [0.00;0.30]	0.00 [0.00;0.10]	0.04**	0.217
RvD1$_{n\text{-}3\ DPA}$		0.20 [0.10;0.30]	0.20 [0.10;0.30]	0.10 [0.00;0.23]	0.337	0.568
RvD2$_{n\text{-}3\ DPA}$		0.80 [0.48;1.10]	0.30 [0.20;0.60]	0.25 [0.08;0.43]	<0.001**	<0.001**
RvD5 $_{n\text{-}3\ DPA}$	Negative	30 (68.2%)	28 (59.6%)	11 (91.7%)	0.447	0.619
	Positive	14 (31.8%)	19 (40.4%)	1 (8.33%)		
PD1 $_{n\text{-}3\ DPA}$		5.60 [0.00;13.6]	0.30 [0.00;7.25]	0.35 [0.00;2.60]	0.156	0.398
10S,17S-diHDPA	Negative	28 (63.6%)	35 (74.5%)	6 (50.0%)	0.861	0.968
	Positive	16 (36.4%)	12 (25.5%)	6 (50.0%)		
MaR1$_{n\text{-}3\ DPA}$	Negative	27 (61.4%)	31 (66.0%)	11 (91.7%)	0.09	0.311
	Positive	17 (38.6%)	16 (34.0%)	1 (8.33%)		
7S,14S-diHDPA		0.80 [0.00;1.25]	0.70 [0.00;1.30]	0.05 [0.00;0.48]	0.374	0.577
**EPA metabolome**						
RvE1		0.85 [0.38;1.60]	0.50 [0.05;1.60]	0.30 [0.08;1.12]	0.091	0.311
RvE2		4.90 [3.75;6.43]	5.50 [3.80;7.90]	4.70 [2.62;6.30]	0.975	0.993
RvE3	Negative	29 (65.9%)	30 (63.8%)	10 (83.3%)	0.447	0.619
	Positive	15 (34.1%)	17 (36.2%)	2 (16.7%)		
**AA metabolome**						
LXA$_4$		0.10 [0.00;0.10]	0.10 [0.00;0.10]	0.10 [0.00;0.10]	0.533	0.702
LXB$_4$		3.80 [1.85;14.5]	5.20 [1.45;36.2]	6.60 [2.95;21.2]	0.404	0.59
5S,15S-diHETE		6.10 [1.05;10.8]	4.40 [1.90;7.90]	6.35 [3.93;12.4]	0.967	0.993
15-epi-LXA$_4$		0.50 [0.30;0.70]	0.20 [0.10;0.40]	0.40 [0.18;0.52]	0.005**	0.05
15-epi-LXB$_4$		77.2 [58.4;88.7]	16.4 [0.25;36.6]	0.30 [0.08;15.6]	<0.001**	<0.001**
LTB$_4$		3.60 [1.37;12.2]	6.50 [2.45;16.7]	2.05 [0.40;5.50]	0.805	0.925
5S, 12S-diHETE	Negative	39 (88.6%)	38 (80.9%)	8 (66.7%)	0.076	0.311
	Positive	5 (11.4%)	9 (19.1%)	4 (33.3%)		
12-epi-LTB$_4$		1.05 [0.50;3.32]	1.60 [0.45;3.00]	0.30 [0.27;1.20]	0.363	0.577
6-trans, 12-epi-LTB$_4$		1.10 [0.50;3.35]	1.50 [0.50;3.00]	0.30 [0.20;1.50]	0.277	0.516
20-OH-LTB$_4$		1.00 [0.18;5.65]	2.70 [0.35;9.25]	3.10 [0.70;9.18]	0.162	0.398
LTC$_4$	Negative	27 (61.4%)	22 (46.8%)	5 (41.7%)	0.125	0.356
	Positive	17 (38.6%)	25 (53.2%)	7 (58.3%)		
LTD$_4$		0.80 [0.30;2.82]	0.80 [0.00;4.95]	0.60 [0.00;3.20]	0.733	0.921
LTE$_4$		65.3 [38.3;188]	156 [47.6;456]	185 [75.4;1943]	0.018**	0.139
PGD$_2$		3.15 [0.55;4.23]	2.40 [0.90;6.45]	4.55 [2.08;10.3]	0.166	0.398
PGE$_2$		35.1 [9.55;108]	61.6 [28.1;207]	72.0 [21.5;162]	0.051	0.228
PGF$_2$□		72.2 [37.7;157]	137 [67.0;387]	179 [55.2;253]	0.032**	0.204
TxB$_2$		46.5 [12.6;70.3]	54.6 [23.5;93.6]	60.0 [12.1;110]	0.215	0.446

All summary statistics are absolute counts (%) for categorical variables and median [inter-quartile range] for continuous data. n refers to the number of patients with non-missing data for the corresponding variable. *Negative means lipid measurement under the detection limit. Positive means lipid measurement above the detection limit.** Note that p value <0.05

Supplementary Table 3: Correlation between the concentrations of select lipid mediator concentrations in the CSF and disease severity

Lipid Mediators	Frequency*	OR (2 vs 1)	95%CI (2 vs 1)	OR (3 vs 1)	95%CI (3 vs 1)
15-epi-LXB_4	1000	0.70	[0.61 ;0.81]	0.63	[0.49 ;0.82]
LXB_4	1000	1.02	[0.99 ;1.03]	1.00	[0.98 ;1.03]
PGE_2	1000	1.05	[1.01 ;1.09]	1.02	[0.97 ;1.08]
22-OH-PD1 (Neg/Pos)	1000	2.50	[0.46 ;13.61]	29.40	[4.74 ;182.3]
PD1	935	2.55	[0.68 ;9.60]	0.24	[0.02 ;3.70]
$RvD5_{n\text{-}3\ DPA}$ (Neg/Pos)	935	1.45	[0.61 ;3.44]	0.19	[0.02 ;1.66]
$RvD2_{n\text{-}3\ DPA}$	830	0.15	[0.05 ;0.50]	0.17	[0.03 ;1.02]
RvE2	830	1.05	[0.92 ;1.20]	0.84	[0.64 ;1.13]
15-epiLXA_4	683	0.07	[0.01 ;0.39]	0.49	[0.09 ;2.74]
LTD_4	683	1.06	[0.98 ;1.15]	0.99	[0.86 ;1.15]
$RvD1_{n\text{-}3\ DPA}$	683	1.76	[0.50 ;6.18]	0.23	[0.01 ;8.85]
20-OH-LTB_4	519	1.00	[0.97 ;1.04]	1.02	[0.99 ;1.06]
MaR1 (Neg/Pos)	519	3.26	[1.31 ;8.08]	4.76	[1.24 ;18.30]
RvD2 (Neg/Pos)	519	0.67	[0.28 ;1.61]	1.13	[0.31 ;4.16]
RvT3 (Neg/Pos)	519	1.50	[0.66 ;3.46]	0.48	[0.11 ;2.03]
10S,17S-diHDPA (Neg/Pos)	369	1.50	[0.75 ;2.98]	0.84	[0.27 ;3.31]
7S,14S-diHDHA (Neg/Pos)	206	0.61	[0.25 ;1.47]	1.13	[0.31 ;4.16]
RvE3 (Neg/Pos)	206	1.10	[0.46 ;2.59]	0.39	[0.07 ;2.00]
LTE_4	105	1.01	[1.00 ;1.02]	1.01	[1.00 ;1.02]
17R-RvD3 (Neg/Pos)	56	0.61	[0.26 ;1.46]	0.13	[0.02 ;1.11]

* High frequency represents the likely chosen as the strong predictors of the disease severity.
CI, confidence interval; OR, odds ratio

Supplemental Table 4: Summary Table Survivors/Non-survivors

Characteristic	n	Summary statistic Survivor (N=95)	n	Summary statistic Non-survivor (N=8)
Gender – no. (%)	95		7	
- Male		62/95 (65%)		2/7 (29%)
- Female		33/95 (35%)		5/7 (71%)
Age (years) – median (IQR)	95	40.00(30.00,49.00)	7	41.00(38.50,49.00)
Weight (kg) – median (IQR)	95	50.00(45.25,60.00)	7	58.50(48.25,60.00)
Glasgow coma score – median (IQR)	95	15.00(13.00,15.00)	8	11.50(8.00,14.00)
*Diagnostic category – no. (%)	95		8	
- definite TBM		76/95 (80%)		5/8 (62%)
- possible TBM		14/95 (15%)		1/8 (12%)
- probable TBM		5/95 (5%)		2/8 (25%)
Treatment arm – no. (%)	95		8	
- Aspirin_1000mg		32/95 (34%)		0/8 (0%)
- Aspirin_81mg		30/95 (32%)		5/8 (62%)
- Placebo		33/95 (35%)		3/8 (38%)

All summary statistics are absolute counts (%) for categorical variables and median (inter-quartile range = IQR) for continuous data. n refers to the number of patients with non-missing data for the corresponding variable.

Supplemental Table 5: Comparison of lipid mediators by survival outcome

Lipid Mediators		Survivors (N=95)	Non-Survivor (N=8)	P-value p-value	Adjusted
**DHA metabolome**					
RvD1	Negative*	59 (62.1%)	5 (62.5%)	1	1
	Positive	36 (37.9%)	3 (37.5%)		
RvD2	Negative	62 (65.3%)	5 (62.5%)	1	1
	Positive	33 (34.7%)	3 (37.5%)		
RvD3	Negative	69 (72.6%)	5 (62.5%)	0.684	0.939
	Positive	26 (27.4%)	3 (37.5%)		
RvD4		0.30 [0.10;0.50]	0.15 [0.00;0.20]	0.077	0.258
RvD5		0.60 [0.20;1.15]	0.10 [0.00;0.23]	0.028**	0.179
RvD6	Negative	62 (65.3%)	6 (75.0%)	0.374	0.939
	Positive	33 (34.7%)	2 (25.0%)		
17R-RvD1	Negative	49 (51.6%)	3 (37.5%)	0.488	0.826
	Positive	46 (48.4%)	5 (62.5%)		
17R-RvD3	Negative	62 (65.3%)	8 (100%)	0.052	0.202
	Positive	33 (34.7%)	0 (0.00%)		
PD1		0.30 [0.00;0.50]	0.00 [0.00;0.08]	0.013**	0.114
10S,17S-diHDHA		0.30 [0.00;0.60]	0.10 [0.00;0.43]	0.374	0.784
17R-PD1	Negative	86 (90.5%)	7 (87.5%)	0.572	0.867
	Positive	9 (9.47%)	1 (12.5%)		
22-OH-PD1	Negative	82 (86.3%)	7 (87.5%)	1	1
	Positive	13 (13.7%)	1 (12.5%)		
PCTR1	Negative	83 (87.4%)	8 (100%)	0.591	0.867
	Positive	12 (12.6%)	0 (0.00%)		
PCTR2	Negative	74 (77.9%)	5 (62.5%)	0.392	0.784
	Positive	21 (22.1%)	3 (37.5%)		
PCTR3	Negative	34 (77.3%)	38 (80.9%)	0.385	0.784
	Positive	10 (22.7%)	9 (19.1%)		
MaR1	Negative	58 (61.1%)	5 (62.5%)	1	1
	Positive	37 (38.9%)	3 (37.5%)		
7S,14S-diHDHA	Negative	27 (61.4%)	34 (72.3%)	0.727	0.939
	Positive	17 (38.6%)	13 (27.7%)		
MaR2		2.20 [0.20;13.8]	0.05 [0.00;0.20]	0.002**	0.05
4S,14S-diHDHA	Negative	56 (58.9%)	6 (75.0%)	0.742	0.826
	Positive	39 (41.1%)	2 (25.0%)		
22-OH-MaR1	Negative	61 (64.2%)	5 (62.5%)	1	1
	Positive	34 (35.8%)	3 (37.5%)		
MCTR1	Negative	88 (92.6%)	8 (100%)	1	1
	Positive	7 (7.37%)	0 (0.00%)		
MCTR2	Negative	92 (96.8%)	8 (100%)	1	1
	Positive	3 (3.16%)	0 (0.00%)		
MCTR3	Negative	81 (85.3%)	8 (100%)	0.594	0.867
	Positive	14 (14.7%)	0 (0.00%)		
**n-3 DPA metabolome**					
RvT1		0.10 [0.05;0.25]	0.15 [0.10;0.20]	0.635	0.902
RvT2		0.40 [0.15;1.20]	0.05 [0.00;0.10]	0.003**	0.05
RvT3	Negative	52 (54.7%)	6 (75.0%)	0.461	0.826
	Positive	43 (45.3%)	2 (25.0%)		
RvT4		0.10 [0.00;0.30]	0.00 [0.00;0.10]	0.05**	0.202
$RvD1_{n-3\ DPA}$		0.20 [0.10;0.30]	0.05 [0.00;0.25]	0.256	0.602
$RvD2_{n-3\ DPA}$		0.50 [0.30;0.80]	0.20 [0.00;0.32]	0.008**	0.09
$RvD5_{n-3\ DPA}$	Negative	61 (64.2%)	8 (100%)	0.05**	0.202
	Positive	34 (35.8%)	0 (0.00%)		
$PD1_{n-3\ DPA}$		0.60 [0.00;9.60]	0.15 [0.00;0.32]	0.086	0.273
10S,17S-diHDPA	Negative	63 (66.3%)	6 (75.0%)	1	1
	Positive	32 (33.7%)	2 (25.0%)		
$MaR1_{n-3\ DPA}$	Negative	64 (67.4%)	5 (62.5%)	1	1
	Positive	31 (32.6%)	3 (37.5%)		
7S,14S-diHDPA		0.70 [0.00;1.30]	0.00 [0.00;0.05]	0.053	0.202
**EPA metabolome**					
RvE1		0.70 [0.20;1.60]	0.15 [0.00;1.02]	0.127	0.361

RvE2		5.10 [3.65;6.50]	5.85 [3.62;6.52]	0.897	1
RvE3	Negative	63 (66.3%)	6 (75.0%)	1	1
	Positive	32 (33.7%)	2 (25.0%)		
**AA metabolome**					
LXA_4		0.10 [0.00;0.10]	0.05 [0.00;0.10]	0.161	0.4334
LXB_4		4.20 [1.80;28.1]	4.65 [2.72;6.45]	0.49	0.826
5S,15S-diHETE		5.40 [1.90;10.2]	7.75 [4.67;8.23]	0.591	0.867
15-epi-LXA_4		0.30 [0.20;0.60]	0.45 [0.35;0.60]	0.515	0.842
15-epiLXB_4		37.5 [10.2;77.3]	0.00 [0.00;0.20]	<0.001**	0.02**
LTB_4		5.00 [1.65;16.7]	2.10 [0.92;3.65]	0.07	0.251
5S,12S-diHETE	Negative	81 (85.3%)	4 (50.0%)	0.03**	0.179
	Positive	14 (14.7%)	4 (50.0%)		
12-epi-LTB_4		1.30 [0.45;3.10]	0.35 [0.20;0.60]	0.037**	0.2
6-trans,12-epi-LTB_4		1.10 [0.45;3.10]	0.35 [0.30;1.30]	0.092	0.276
20-OH-LTB_4		1.40 [0.25;8.60]	1.75 [0.75;5.52]	0.753	0.968
LTC_4	Negative	51 (53.7%)	3 (37.5%)	0.473	0.826
	Positive	44 (46.3%)	5 (62.5%)		
LTD_4		0.80 [0.20;3.05]	1.00 [0.00;11.9]	0.98	1
LTE_4		92.7 [43.3;335]	827 [524;1305]	0.007**	0.09
PGD_2		2.70 [0.90;4.35]	7.10 [5.27;14.0]	0.015	0.116
PGE_2		45.7 [17.5;168]	33.9 [3.38;61.7]	0.198	0.508
PGF□□		111 [48.4;261]	84.6 [22.3;141]	0.229	0.563
TXB_2		48.7 [16.2;85.0]	78.6 [49.9;104]	0.358	0.784

All summary statistics are absolute counts (%) for categorical variables and median [inter-quartile range] for continuous data. n refers to the number of patients with non-missing data for the corresponding variable.

*Negative means lipid measurement under the detection limit. Positive means lipid measurement above the detection limit.

** Note that p value <0.05

Supplementary Table 6: Correlation between select concentrations of lipid mediators in the CSF and mortality

Lipid Mediators	Frequency	OR	95% CI (OR)
PGD_2	998	1.26	[1.10; 1.52]
15-epi-LXB_4	984	0.25	[0.02; 0.67]
17R-RvD3 (Neg/Pos)	974	0.00	NA
5S,12S-diHETE (Neg/Pos)	953	5.78	[1.24; 27.20]
$RvD5_{n\text{-}3\ DPA}$ (Neg/Pos)	938	0.00	NA
PD1	904	0.01	[0.00; 0.37]
PCTR2 (Neg/Pos)	860	2.57	[0.13; 19.22]
RvT3 (Neg/Pos)	785	0.40	[0.06; 1.85]
PCTR1 (Neg/Pos)	695	0.00	NA
MCTR1 (Neg/Pos)	610	0.00	NA
RvD6 (Neg/Pos)	610	0.63	[0.09; 2.89]
PGF_{2}□	528	0.96	[0.87; 1.00]
$RvD1_{n\text{-}3\ DPA}$	456	0.210	[0.00; 2.84]
10S,17S-diHDPA (Neg/Pos)	378	0.66	[0.09; 3.04]
LTC_4 (Neg/Pos)	302	1.93	[0.45; 9.85]
LXB_4	165	0.94	[0.81; 1.00]
MaR1 (Neg/Pos)	165	0.94	[0.18; 4.07]
$PD1_{n\text{-}3\ DPA}$	165	0.57	[0.00; 0.90]
RvT2	115	0.02	[0.00; 0.43]

Supplemental Table 7: Summary table placebo, 81 mg and 1000mg aspirin

Characteristic	n	Summary statistic Placebo (N=36)	n	Summary statistic 81mg aspirin (N=35)	n	Summary statistic 1000mg aspirin (N=32)
Gender – no. (%)	36		34		32	
- Male		24/36 (67%)		24/34 (71%)		16/32 (50%)
- Female		12/36 (33%)		10/34 (29%)		16/32 (50%)
Age (years) – median (IQR)	36	42.00(32.75,50.00)	34	39.00(32.50,47.75)	32	39.50(29.75,51.25)
Weight (kg) – median (IQR)	36	50.75(44.75,60.00)	34	50.50(45.00,58.88)	32	50.00(46.38,60.50)
Glasgow coma score – median (IQR)	36	14.00(12.75,15.00)	35	14.00(12.50,15.00)	32	15.00(12.75,15.00)
Diagnostic category – no. (%)	36		35		32	
- definite TBM		31/36 (86%)		26/35 (74%)		24/32 (75%)
- possible TBM		3/36 (8%)		5/35 (14%)		7/32 (22%)
- probable TBM		2/36 (6%)		4/35 (11%)		1/32 (3%)

All summary statistics are absolute counts (%) for categorical variables and median (inter-quartile range = IQR) for continuous data. n refers to the number of patients with non-missing data for the corresponding variable.

Supplemental Table 8: **Comparison of lipid mediator reduction after 30 days between placebo and 81mg aspirin**

Lipid Mediators	Placebo (N=29) Median [IQR]	Aspirin 81mg (N=27) Median [IQR]	Treatment effect Mean (95% CI)	P value	P Adjusted
**DHA metabolome**					
RvD1	0.00 [0.00;0.20]	0.10 [0.00;0.20]	0.032 (-0.053;0.118)	0.4521	0.7118
RvD2	1.10 [0.20;2.10]	1.20 [0.05;2.85]	0.495 (-0.24;1.23)	0.1825	0.7118
RvD3	0.00 [0.00;0.00]	0.00 [-0.10;0.00]	-0.001 (-0.046;0.044)	0.9657	0.9965
RvD4	-0.20 [-0.40;-0.10]	-0.20 [-0.30;-0.10]	0.309 (-0.288;0.907)	0.3035	0.7118
RvD5	-0.80 [-1.40;-0.20]	-0.40 [-0.80;-0.15]	0.147 (-0.167;0.462)	0.3512	0.7118
RvD6	0.00 [0.00;0.10]	0.00 [0.00;0.10]	-0.025 (-0.186;0.136)	0.7573	0.9074
17R_RvD1	0.00 [-0.10;0.00]	0.00 [0.00;0.00]	-0.001 (-0.052;0.05)	0.9712	0.9965
17R_RvD3	0.00 [-0.10;0.00]	0.00 [-0.05;0.00]	0.008 (-0.023;0.038)	0.6141	0.8022
PD1	-0.10 [-0.30;0.10]	-0.10 [-0.20;0.10]	0.022 (-0.129;0.173)	0.7678	0.9074
10S,17S-diHDHA	-0.40 [-0.50;0.00]	-0.30 [-0.80;0.00]	0.328 (-0.388;1.045)	0.3617	0.7118
17R-PD1	0.00 [0.00;0.00]	0.00 [0.00;0.00]	0.006 (-0.053;0.066)	0.8287	0.9368
22-OH-PD1	0.40 [0.20;0.70]	0.30 [0.00;0.65]	0.202 (-0.33;0.734)	0.4496	0.7118
PCTR1	0.00 [0.00;0.00]	0.00 [0.00;0.00]	0.191 (-0.093;0.476)	0.1833	0.7118
PCTR2	0.00 [0.00;0.00]	0.00 [0.00;0.00]	-0.042 (-0.129;0.045)	0.3369	0.7118
PCTR3	0.00 [-0.20;0.00]	0.00 [0.00;0.00]	1.24 (-0.682;3.161)	0.2013	0.7118
MaR1	0.00 [-0.10;0.10]	0.00 [-0.10;0.10]	-0.082 (-0.229;0.064)	0.2659	0.7118
7S,14S-diHDHA	0.00 [-1.00;0.00]	0.00 [-0.90;0.15]	1.097 (-0.809;3.004)	0.2534	0.7118
MaR2	-1.80 [-11.00;0.20]	-1.10 [-9.55;0.35]	-2.071 (-7.048;2.905)	0.4076	0.7118
4S,14S-diHDHA	0.00 [-0.30;0.00]	0.00 [-0.20;0.00]	-0.004 (-0.036;0.029)	0.8122	0.9368
22-OH-MaR1	0.30 [0.00;0.80]	0.10 [-0.10;0.60]	-0.125 (-0.521;0.271)	0.5293	0.7864
MCTR1	0.00 [0.00;0.00]	0.00 [0.00;0.00]	NA	NA	NA
MCTR2	NA	NA	NA	NA	NA
MCTR3	0.00 [0.00;0.00]	0.00 [0.00;0.00]	-0.776 (-2.574;1.021)	0.3903	0.7118
**n-3 DPA metabolome**					
RvT1	0.00 [-0.10;0.00]	0.00 [-0.20;0.10]	0.025 (-0.078;0.128)	0.6261	0.8022
RvT2	-0.10 [-0.40;0.10]	-0.20 [-0.75;0.15]	-0.09 (-0.459;0.278)	0.6245	0.8022
RvT3	0.00 [-0.10;0.00]	0.00 [-0.10;0.00]	0.012 (-0.012;0.037)	0.3267	0.7118
RvT4	-0.20 [-0.30;0.00]	-0.10 [-0.20;0.00]	-0.019 (-0.068;0.029)	0.4221	0.7118
$RvD1_{n-3DPA}$	-0.10 [-0.20;0.00]	-0.10 [-0.30;-0.05]	-0.012 (-0.073;0.05)	0.7017	0.8688
$RvD2_{n-3\ DPA}$	-0.60 [-0.90;-0.30]	-0.40 [-0.80;-0.20]	-0.035 (-0.117;0.047)	0.3979	0.7118
$RvD5_{n-3\ DPA}$	0.00 [-0.50;0.00]	0.00 [0.00;0.20]	0.139 (-0.078;0.356)	0.2051	0.7118
$PD1_{n-3DPA}$	0.20 [-4.30;3.10]	0.60 [-4.00;4.55]	3.535 (-4.322;11.392)	0.3709	0.7118
10S,17S-diHDPA	3.80 [0.00;8.10]	1.60 [0.00;5.30]	-2.377 (-5.831;1.077)	0.1732	0.7118
MaR1n-3DPA	0.00 [-0.80;0.20]	0.00 [0.00;0.45]	0.467 (-0.006;0.94)	0.0529	0.7118
7S,14S-diHDPA	0.00 [-1.00;1.20]	0.20 [-0.35;1.10]	0.259 (-0.82;1.338)	0.6325	0.8022
**EPA metabolome**					
RvE1	-0.80 [-1.40;-0.20]	-0.40 [-1.40;-0.20]	0.224 (-0.3;0.748)	0.3953	0.7118
RvE2	-2.30 [-4.30;-0.20]	-1.40 [-2.60;-0.25]	3.439 (-1.757;8.635)	0.1901	0.7118
RvE3	0.00 [0.00;0.10]	0.00 [-0.30;0.00]	-0.007 (-0.482;0.468)	0.9763	0.9965
**AA metabolome**					
LXA_4	-0.10 [-0.10;0.00]	-0.10 [-0.10;0.00]	-0.008 (-0.034;0.019)	0.5551	0.8018
LXB_4	-6.60 [-27.30;1.50]	-3.40 [-42.60;1.05]	0.029 (-2.872;2.929)	0.9843	0.9965
5S,15S-diHETE	-0.10 [-8.50;2.50]	0.00 [-4.20;1.25]	1.96 (-3.387;7.307)	0.4654	0.7118
15-epi-LXA_4	-0.20 [-0.40;0.00]	-0.30 [-0.50;-0.10]	0 (-0.151;0.152)	0.9965	0.9965
15-epiLXB_4	-9.90 [-28.00;-1.40]	-15.90 [-47.25;-1.75]	-4.4 (-15.714;6.915)	0.4389	0.7118
LTB_4	-1.40 [-14.50;0.10]	-2.30 [-8.65;-0.10]	3.666 (-4.59;11.921)	0.3772	0.7118
5S,12S-diHETE	0.00 [0.00;0.00]	0.00 [0.00;0.00]	-0.095 (-0.328;0.137)	0.4145	0.7118
12-epi-LTB_4	-1.00 [-2.90;0.00]	-0.80 [-2.95;-0.10]	0.742 (-0.649;2.134)	0.2896	0.7118
6-trans,12-epi-LTB_4	-1.30 [-2.80;-0.10]	-0.80 [-2.90;-0.05]	0.736 (-0.396;1.869)	0.198	0.7118
20-OH-LTB_4	8.60 [-0.70;32.7]	10.7 [5.70;26.4]	-3.303 (-16.5;9.893)	0.6177	0.8022
LTC_4	0.00 [-0.20;0.10]	0.00 [-0.25;0.00]	0.873 (-0.789;2.536)	0.297	0.7118
LTD_4	0.00 [-1.70;0.50]	-0.10 [-1.10;0.75]	5.204 (-5.802;16.211)	0.3472	0.7118
LTE_4	-49.80 [-142.70;-35.90]	-41.40 [-87.80;-11.15]	33.99 (-32.565;100.554)	0.3103	0.7118
PGD_2	0.10 [-0.50;2.20]	0.30 [-1.80;1.85]	-0.173 (-2.472;2.127)	0.8809	0.9746
PGE_2	-36.80 [-147.80;-7.80]	-25.30 [-123.40;-2.25]	-10.589 (-26.341;5.162)	0.1833	0.7118
PGF_{2a}	-52.30 [-289.70;-20.80]	-74.80 [-169.90;-11.60]	-17.393 (-44.631;9.845)	0.2059	0.7118
TxB_2	-14.30 [-39.20;1.80]	-22.90 [-70.85;-6.50]	-5.27 (-18.225;7.685)	0.4182	0.7118

All summary statistics are absolute counts (%) for categorical variables and median (inter-quartile range = IQR) for continuous data, and mean and 95% CI for lipid mediator reduction measurement. n refers to the number of patients with non-missing data for the corresponding variable. ** Note that p value <0.05

Supplemental Table 9: Comparison of lipid mediator reduction after 30 days between placebo and 1000mg aspirin

Lipid Mediators	Placebo (N=29) Median [IQR]	Aspirin 1000mg (N=23) Median [IQR]	Treatment effect Mean (95% CI)	P-value	Adjusted p-value
**DHA metabolome**					
RvD1	0.00 [0.00;0.20]	0.00 [0.00;0.15]	-0.042 (-0.113;0.028)	0.2298	0.7531
RvD2	1.10 [0.20;2.10]	1.80 [0.25;2.95]	0.547 (-0.155;1.248)	0.1239	0.7531
RvD3	0.00 [0.00;0.00]	0.00 [0.00;0.10]	0.022 (-0.01;0.054)	0.1736	0.7531
RvD4	-0.20 [-0.40;-0.10]	-0.20 [-0.50;-0.10]	0.011 (-0.053;0.074)	0.7345	0.879
RvD5	-0.80 [-1.40;-0.20]	-0.50 [-2.15;-0.25]	0.071 (-0.03;0.173)	0.1636	0.7531
RvD6	0.00 [0.00;0.10]	0.00 [-0.20;0.10]	-0.019 (-0.098;0.06)	0.6338	0.8749
17R_RvD1	0.00 [-0.10;0.00]	0.00 [-0.20;0.00]	-0.021 (-0.073;0.031)	0.4274	0.7531
17R_RvD3	0.00 [-0.10;0.00]	0.00 [-0.05;0.00]	0.005 (-0.018;0.027)	0.687	0.879
PD1	-0.10 [-0.30;0.10]	-0.10 [-0.35;0.20]	0.049 (-0.086;0.185)	0.4698	0.7531
10S,17S-diHDHA	-0.40 [-0.50;0.00]	-0.20 [-0.50;0.05]	0.043 (-0.08;0.167)	0.4831	0.7531
17R-PD1	0.00 [0.00;0.00]	0.00 [0.00;0.00]	-0.005 (-0.04;0.031)	0.7919	0.893
22-OH-PD1	0.40 [0.20;0.70]	0.40 [0.35;0.65]	0.111 (-0.061;0.284)	0.2007	0.7531
PCTR1	0.00 [0.00;0.00]	0.00 [0.00;0.00]	0.064 (-0.012;0.14)	0.0988	0.7531
PCTR2	0.00 [0.00;0.00]	0.00 [0.00;0.00]	0.117 (-0.121;0.355)	0.3276	0.7531
PCTR3	0.00 [-0.20;0.00]	0.00 [-0.35;0.30]	0.231 (-0.466;0.929)	0.5083	0.7685
MaR1	0.00 [-0.10;0.10]	0.00 [-0.05;0.15]	-0.089 (-0.237;0.06)	0.2349	0.7531
7S,14S-diHDHA	0.00 [-1.00;0.00]	0.00 [-2.00;0.00]	0.234 (-0.414;0.882)	0.4711	0.7531
MaR2	-1.80 [-11.00;0.20]	-1.80 [-7.55;0.35]	1.236 (-4.928;7.401)	0.6887	0.879
4S,14S-diHDHA	0.00 [-0.30;0.00]	-0.30 [-0.45;0.00]	-0.003 (-0.04;0.034)	0.8831	0.9214
22-OH-MaR1	0.30 [0.00;0.80]	0.40 [0.00;0.80]	0.006 (-0.404;0.415)	0.9771	0.9771
MCTR1	0.00 [0.00;0.00]	0.00 [0.00;0.00]	0.146 (-0.037;0.33)	0.1151	0.7531
MCTR2	NA	NA	NA	NA	NA
MCTR3	0.00 [0.00;0.00]	0.00 [-0.75;0.00]	-0.874 (-2.898;1.149)	0.3894	0.7531
**n-3 DPA metabolome**					
RvT1	0.00 [-0.10;0.00]	-0.10 [-0.20;0.00]	-0.031 (-0.097;0.036)	0.357	0.7531
RvT2	-0.10 [-0.40;0.10]	-0.20 [-1.30;0.00]	-0.047 (-0.484;0.39)	0.8307	0.9141
RvT3	0.00 [-0.10;0.00]	0.00 [-0.10;0.00]	0.018 (-0.008;0.044)	0.1674	0.7531
RvT4	-0.20 [-0.30;0.00]	-0.10 [-0.25;0.00]	0.042 (-0.034;0.117)	0.2697	0.7531
$RvD1_{n-3DPA}$	-0.10 [-0.20;0.00]	-0.20 [-0.30;-0.05]	-0.013 (-0.082;0.055)	0.7024	0.879
$RvD2_{n-3\ DPA}$	-0.60 [-0.90;-0.30]	-0.60 [-0.90;-0.35]	-0.05 (-0.133;0.034)	0.2379	0.7531
$RvD5_{n-3\ DPA}$	0.00 [-0.50;0.00]	0.00 [-0.50;0.05]	0.242 (-0.055;0.539)	0.1085	0.7531
$PD1_{n-3DPA}$	0.20 [-4.30;3.10]	0.10 [-4.55;4.60]	0.478 (-2.832;3.789)	0.7727	0.8903
10S,17S-diHDPA	3.80 [0.00;8.10]	6.00 [1.65;10.3]	1.881 (-2.218;5.98)	0.3611	0.7531
MaR1n-3DPA	0.00 [-0.80;0.20]	0.00 [-0.65;0.00]	0.014 (-0.183;0.211)	0.8866	0.9214
7S,14S-diHDPA	0.00 [-1.00;1.20]	1.00 [-0.55;1.85]	0.403 (-0.606;1.412)	0.4257	0.7531
**EPA metabolome**					
RvE1	-0.80 [-1.40;-0.20]	-1.10 [-1.60;-0.35]	0.022 (-0.201;0.245)	0.8451	0.9141
RvE2	-2.30 [-4.30;-0.20]	-3.20 [-4.80;0.15]	0.649 (-0.822;2.121)	0.3796	0.7531
RvE3	0.00 [0.00;0.10]	0.00 [-0.55;0.00]	-0.055 (-0.179;0.068)	0.3704	0.7531
**AA metabolome**					
LXA_4	-0.10 [-0.10;0.00]	-0.10 [-0.10;0.00]	-0.001 (-0.03;0.028)	0.9473	0.9655
LXB_4	-6.60 [-27.30;1.50]	-4.40 [-22.45;0.25]	-0.984 (-3.776;1.808)	0.4822	0.7531
5S,15S-diHETE	-0.10 [-8.50;2.50]	-2.70 [-7.45;1.75]	-0.598 (-2.575;1.38)	0.5463	0.7825
15-epi-LXA_4	-0.20 [-0.40;0.00]	-0.20 [-0.55;-0.10]	0.016 (-0.08;0.112)	0.7463	0.879
15-epiLXB_4	-9.90 [-28.00;-1.40]	-42.80 [-53.50;-13.70]	-5.798 (-16.873;5.277)	0.2979	0.7531
LTB_4	-1.40 [-14.50;0.10]	-2.70 [-28.55;0.70]	10.529 (-1.997;23.055)	0.0975	0.7531
5S,12S-diHETE	0.00 [0.00;0.00]	0.00 [0.00;0.00]	-0.088 (-0.321;0.145)	0.4503	0.7531
12-epi-LTB_4	-1.00 [-2.90;0.00]	-1.30 [-3.70;0.20]	1.444 (0.017;2.871)	0.0475	0.7531
6-trans,12-epi-LTB_4	-1.30 [-2.80;-0.10]	-1.30 [-3.75;0.15]	1.087 (0.076;2.097)	0.0356	0.7531
20-OH-LTB_4	8.60 [-0.70;32.7]	16.9 [4.95;25.1]	2.918 (-9.683;15.518)	0.6438	0.8749
LTC_4	0.00 [-0.20;0.10]	0.00 [-0.20;0.10]	-0.05 (-0.34;0.239)	0.729	0.879
LTD_4	0.00 [-1.70;0.50]	-0.30 [-1.85;0.40]	-1.432 (-5.895;3.031)	0.522	0.7685
LTE_4	-49.80 [-142.70;-35.90]	-63.40 [-151.40;-33.40]	-14.622 (-51.896;22.653)	0.4343	0.7531
PGD_2	0.10 [-0.50;2.20]	0.70 [-2.70;3.55]	-0.76 (-2.174;0.653)	0.2852	0.7531
PGE_2	-36.80 [-147.80;-7.80]	-66.10 [-190.95;0.60]	-6.488 (-24.774;11.797)	0.4792	0.7531
PGF_{2a}	-52.30 [-289.70;-20.80]	-101.10 [-234.90;-6.90]	-13.878 (-44.256;16.5)	0.3631	0.7531
TxB_2	-14.30 [-39.20;1.80]	-42.10 [-74.35;-5.75]	-19.353 (-28.317;-10.389)	0.0001	0.0053

All summary statistics are absolute counts (%) for categorical variables and median (inter-quartile range = IQR) for continuous data, and mean and 95% CI for lipid mediator reduction measurement. n refers to the number of patients with non-missing data for the corresponding variable. ** Note that p.value <0.05

Supplemental Figures

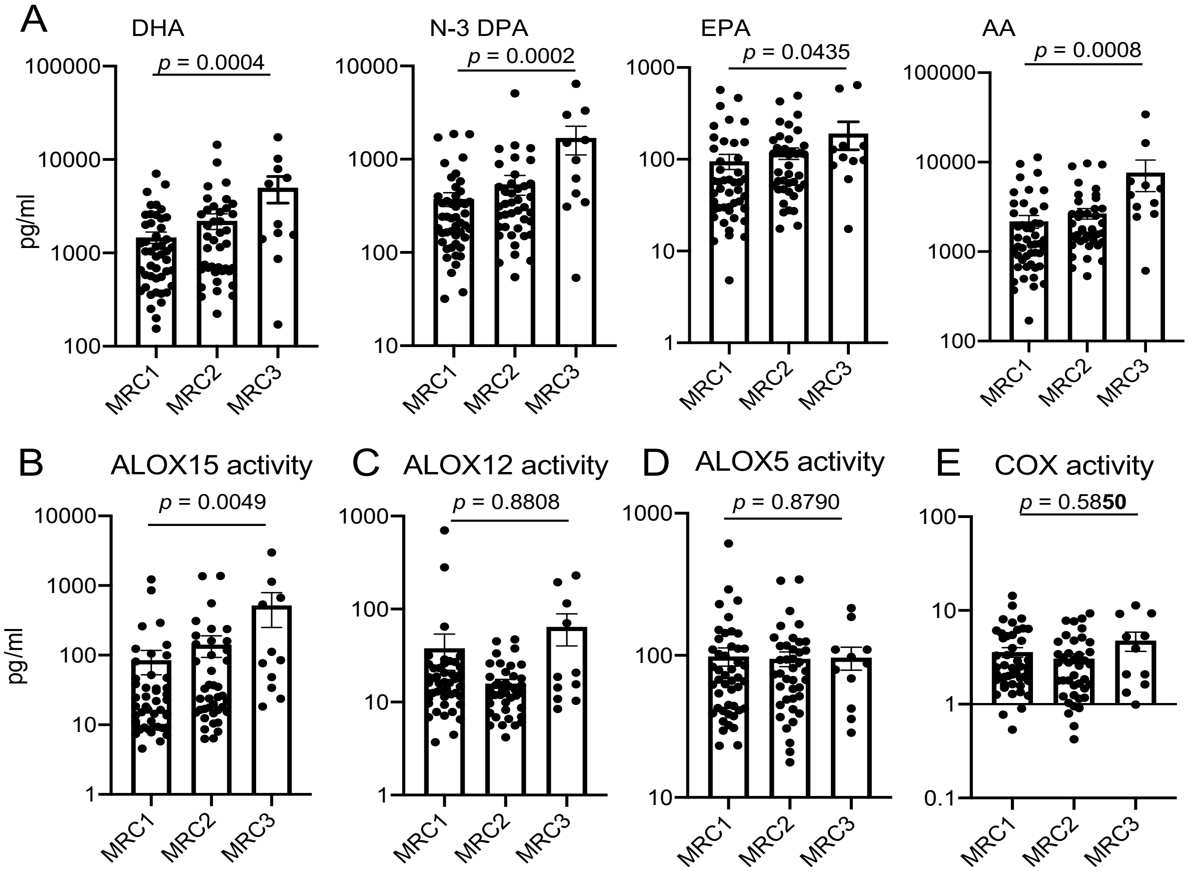

Supplemental Figure 1: Increased free fatty acid concentrations and ALOX15 products with increasing disease severity. CSF were collected from patients with TBM before the start of treatment and products were extracted, identified and quantified using lipid mediator profiling. (A) free fatty acid concentrations. (B-E) summation of (B) ALOX15 products - 17-HDHA, 17-HDPA, 15-HEPE, 15-HETE (C) ALOX12 products – 14-HDHA, 14-HDPA, 12-HEPE, 12-HETE (D) ALOX5 products – 7-HDHA, 7-HDPA, 5-HEPE, 5-HETE (E) COX products - 13-HDHA, 13-HDPA. Statistical differences were determined using one-way ANOVA followed by test for linear trend. Results are mean ± SEM. n = 44 for MRC 1; n = 47 for MRC2 and n = 12 for MRC3.

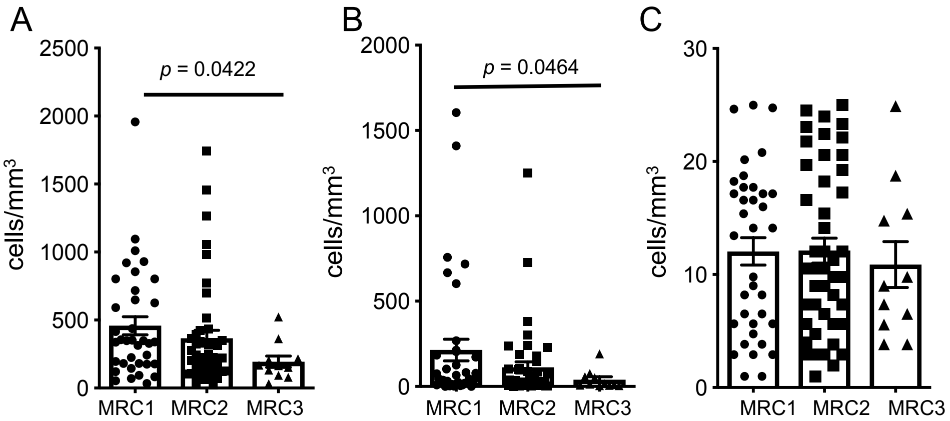

Supplemental Figure 2: Decreased neutrophil counts with increasing disease severity. CSF (A) white blood cell, (B) neutrophil (C) lymphocyte counts were determined prior to treatment initiation. Differences between the different groups were determined using one-way

ANOVA followed by test for linear trend; * p < 0.05. Results are mean ± SEM. n = 44 for MRC1; n = 47 for MRC2 and n = 12 for MRC3.

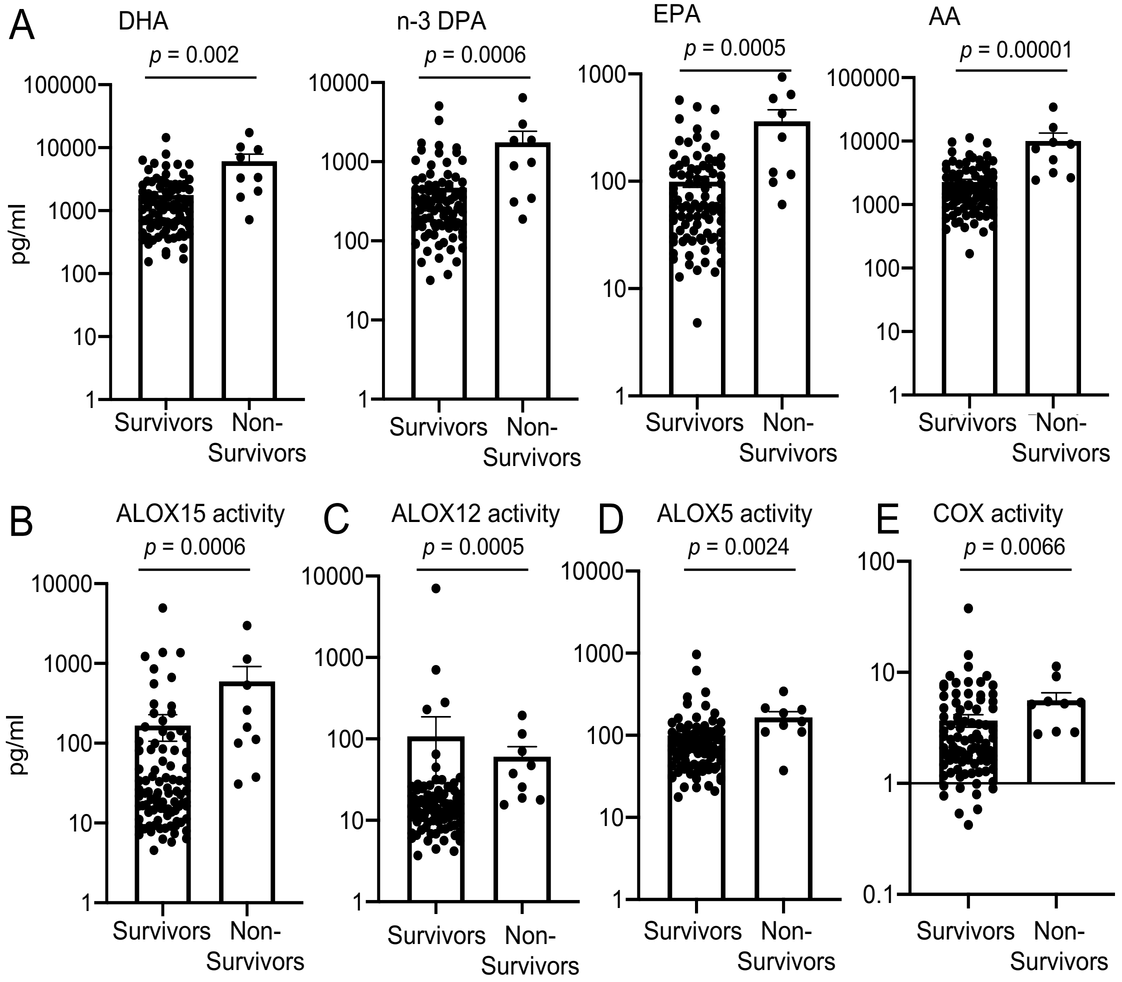

Supplemental Figure 3: Increased free fatty acid concentrations together ALOX and COX products with in TBM non-survivors. CSF were collected from patients with TBM before the start of treatment and products were extracted, identified and quantified using lipid mediator profiling. (A) free fatty acid concentrations. (B-E) summation of (B) ALOX15 products - 17-HDHA, 17-HDPA, 15-HEPE, 15-HETE (C) ALOX12 products – 14-HDHA, 14-HDPA, 12-HEPE, 12-HETE (D) ALOX5 products – 7-HDHA, 7-HDPA, 5-HEPE, 5-HETE (E) COX products- 13-HDHA, 13-HDPA. Statistical different groups were determined using one-way ANOVA followed by test for linear trend. Results are mean ± SEM. n = 95 survivors and 8 non-survivors
